# Longitudinal telomere dynamics within natural lifespans of a wild bird

**DOI:** 10.1038/s41598-023-31435-9

**Published:** 2023-03-15

**Authors:** Michael Le Pepke, Thomas Kvalnes, Jonathan Wright, Yimen G. Araya-Ajoy, Peter Sjolte Ranke, Winnie Boner, Pat Monaghan, Bernt-Erik Sæther, Henrik Jensen, Thor Harald Ringsby

**Affiliations:** 1 Department of Biology, Centre for Biodiversity Dynamics (CBD), Norwegian University of Science and Technology (NTNU), Trondheim, Norway; 2 School of Biodiversity, One Health and Veterinary Medicine, University of Glasgow, Glasgow, UK

**Keywords:** Ecological genetics, Ecophysiology, Evolutionary ecology, Molecular ecology, Evolutionary biology, Heritable quantitative trait, Telomeres, Ageing

## Abstract

Telomeres, the nucleotide sequences that protect the ends of eukaryotic chromosomes, shorten with each cell division and telomere loss may be influenced by environmental factors. Telomere length (TL) decreases with age in several species, but little is known about the sources of genetic and environmental variation in the change in TL (∆TL) in wild animals. In this study, we tracked changes in TL throughout the natural lifespan (from a few months to almost 9 years) of free-living house sparrows (*Passer* *domesticus*) in two different island populations. TL was measured in nestlings and subsequently up to four times during their lifetime. TL generally decreased with age (senescence), but we also observed instances of telomere lengthening within individuals. We found some evidence for selective disappearance of individuals with shorter telomeres through life. Early-life TL positively predicted later-life TL, but the within-individual repeatability in TL was low (9.2%). Using genetic pedigrees, we found a moderate heritability of ∆TL (*h*^2^ = 0.21), which was higher than the heritabilities of early-life TL (*h*^2^ = 0.14) and later-life TL measurements (*h*^2^ = 0.15). Cohort effects explained considerable proportions of variation in early-life TL (60%), later-life TL (53%), and ∆TL (37%), which suggests persistent impacts of the early-life environment on lifelong telomere dynamics. Individual changes in TL were independent of early-life TL. Finally, there was weak evidence for population differences in ∆TL that may be linked to ecological differences in habitat types. Combined, our results show that individual telomere biology is highly dynamic and influenced by both genetic and environmental variation in natural conditions.

## Introduction

Telomeres are short DNA repeats that protect the ends of linear chromosomes^1^. Telomere length (TL) decreases due to incomplete end replication during cell division^2^, and telomere loss can be accelerated by oxidative stress (^3–5^, but see^6^). When telomeres become critically short, replicative cell senescence may be induced^7^. Consequently, telomeres are implicated in organismal senescence^8^ and TL is considered a hallmark of ageing^9^. However, TL or the change in TL (∆TL) is often found to be independent of chronological age^10^, but may be influenced by several environmental factors and experiences^11–13^. For instance, telomeres may shorten in response to efforts associated with reproduction^14,15^, growth^16^, or harsh abiotic conditions in free-living populations^17^. Consequently, TL may be a causal mediator of effects of growth and early-life conditions on later-life senescence^18,19^, and TL may predict fitness components such as survival and reproductive success in wild animals^20,21^.

TL of somatic cells decreases over lifetimes in many vertebrate species^22^, but there are exceptions to this pattern across vertebrates^23–25^. Some studies suggest that most telomere loss occurs during early life^22,26–28^. There are also indications that stress experienced early in life, such as that associated with changes in the tempo of growth, has delayed consequences for later-life telomere shortening^18^ and oxidative stress^29^. However, many studies have been restricted to the use of single cross-sectional TL measurements, particularly in early-life studies, which can be influenced by selective loss of phenotypes at later ages^30^. Early-life TL has been shown to correlate with TL later in life in some species^31,32^. However, there are also studies suggesting that telomere shortening rates are greater in individuals with initially longer telomeres^27,33–35^ perhaps because longer telomeres present a larger target for oxidative damage^36–39^. Such an effect may shape the observed associations between early-life TL, stress exposure and fitness^19,40,41^.

Heritability estimates of TL vary greatly across species and populations^30,42–44^. However, if TL is only measured in adults, it is not clear whether the estimated heritability reflects additive genetic effects (*V*_*A*_) of TL itself versus *V*_*A*_ of individual susceptibility to telomere shortening during their lifetime prior to TL measurement, or both. Indeed, little is known about the heritability of telomere shortening rates. Hjelmborg et al.^45^ estimated a heritability of telomere shortening of *h*^2^ = 0.28 in adult human twins (*n* = 652), which was smaller than their estimated heritability for adult TL (*h*^2^ = 0.64) and TL heritabilities reported in other human studies (e.g.^46^). In contrast, Bauch et al.^47^ found a low heritability of telomere shortening rates (*h*^2^ = 0.09) during the first month of life in western jackdaws (*Coloeus* *monedula*, *n* = 474), but a high heritability of early-life TL (*h*^2^ = 0.74).

In this longitudinal study, we use 24 years of blood sampling (*n* = 3061) from two insular house sparrow (*Passer* *domesticus*) populations to track changes in individual TL throughout natural lifespans. First, we investigate how TL changes with age within individuals from the nestling stage to 9 years of age. In a previous cross-sectional study in these house sparrow populations, we found some evidence for a negative association between TL and age among 5–14 days old nestlings (*n* = 2662^43^). In other house sparrow populations, and with a smaller (cross-sectional) sample size, we did not detect trends in TL with age among 5–17 days old nestlings (*n* = 566^48^). Second, we examine within-individual consistency in TL. Third, we decompose genetic and environmental contributions to variation in ∆TL, early-life and later-life TL. Fourth, we test whether early-life TL is associated with changes in TL. That is, whether individuals with initially longer telomeres also experience more TL shortening. Finally, we explore factors affecting ∆TL through life in the two populations. These populations differ in the values of several key life-history traits^49^ and we have previously found differences in the associations between environmental conditions and early-life TL of these two populations^50^. Furthermore, both TL and ∆TL may be sex-specific in some species^51^ and TL may be negatively associated with body size in house sparrows^43,48^ and in other species^16^. We therefore also test for differences between the two populations in ∆TL and for effects of sex and body size on ∆TL.

## Methods

### Study system

This study involved two unmanipulated island populations of house sparrows in an archipelago in northern Norway that are part of a metapopulation study (see map in^50^). Birds were monitored on Hestmannøy (66° 33′ N, 12° 50′ E) from 1994 to 2020 and on Træna (66° 30′ N, 12°05′ E) from 2004 to 2020. The house sparrow is a small, globally distributed passerine that lives naturally in close association with human habitation, and human activities provide the natural basis of existence for house sparrows^52^. The average population generation time and lifespan in populations similar to those in this study is about 2 years^53^, with a maximum recorded lifespan for this species of 20 years in the wild^54^. On Hestmannøy, the sparrows live mainly on dairy farms, and on Træna they live mainly in gardens in a small village. Nests in cavities inside buildings and other human-made structures (mainly on Hestmannøy) or in nest boxes (on both islands) were visited regularly during the breeding season (May–August) from 1994 to 2013 to ring fledglings with a unique color-ring combination at around 10 days of age (5–14 days). Nestling tarsometatarsus (tarsus) was measured using calipers to nearest 0.01 mm. We estimated age-standardized nestling tarsus length as the residuals of a linear regression of tarsus length on age and age squared separately for each sex and population. Juveniles and adults were captured using mist nets mainly during summer and autumn (May to October) from 1994 to 2020. A small blood sample (25 μL) was collected from all nestlings and recaptured juveniles and adults via venipuncture of the brachial vein. Blood was stored in 96% ethanol at room temperature in the field and at −20 °C in the laboratory prior to DNA extraction as described in Pepke et al.^48^. The study was carried out with permits from the Norwegian Animal Research Authority (FOTS id 11904) and the Ringing Centre at Stavanger Museum, Norway.

### Telomere length measurements

Relative erythrocyte telomere lengths (TL) were measured using the real-time quantitative polymerase chain reaction (qPCR) amplification method^55,56^ as described in Pepke et al.^48^. DNA was extracted from blood using the ReliaPrep Large Volume HT gDNA Isolation System (Promega). DNA concentration was measured using a FLUROostar Omega scanner (BMG Labtech) and diluted with dH2O to yield 1.67 ng/mL, corresponding to 10 ng of DNA per well, and stored at −78 °C. All samples had a 260/280 absorbance ratio of 1.8–2.2 and DNA concentration > 15 ng μl^−1^. Telomeric sequence was measured relative to the amount of the non-variable gene GAPDH and a reference sample. Primers, qPCR assay setup and thermal profiles are described in detail in Pepke et al.^48^. Assays were prepared with the Absolute blue qPCR SYBR green Low Rox master mix (ThermoFisher scientific). A two-fold serial dilution was included on all plates to make a standard curve. Samples were randomized across qPCR plates and run in triplicates, and details of these qPCR runs and efficiencies (all plates within 100 ± 10%, mean telomere and GAPDH assay efficiencies were 97.5 ± 3.9% and 97.6 ± 4.2%, respectively) are given in Pepke et al.^43^. All reactions were carried out by the same person (MLP). Average reference sample cycle thresholds across all plates were 10.54 ± 0.03 SD and 21.53 ± 0.02 SD for telomere and GAPDH assays, respectively. DNA re-extractions followed by runs on different plates revealed highly correlated TL measurements (*R*^2^ = 0.75, see details in Pepke et al.^43^). Data were analyzed using qBASE^57^ while controlling for differences in amplification efficiency between plates and inter-run variation.

Early-life TL was obtained for 2746 nestlings (*n* = 2110 from Hestmannøy and *n* = 636 from Træna) aged 5–14 days old (see Table S1 in the Supporting Information for sample size details). In addition, 228 (*n* = 195 from Hestmannøy and *n* = 33 from Træna) individuals were blood sampled at least once as juveniles and/or adults, providing 315 additional later-life TL measurements. 223 of these individuals had also been sampled as nestlings. The longitudinal data set consists of 226 individuals that were sampled at least twice (165 individuals were sampled twice, 44 sampled thrice, 11 sampled four times and 6 sampled five times), with the number of TL samples taken ranging from 2 to 5 (mean 2.4 ± 0.7) samples per individual (536 samples in total). The time interval between first and last TL measurements ranged from 15 days and up to 3245 days (9 years, Fig. S1). The total number of TL samples was 3061 from 2751 individuals.

### Pedigree information

Molecular sex determination and microsatellite pedigree construction for these populations are described in Jensen et al.^53^ and Billing et al.^58^. House sparrows are socially monogamous, but extra-pair paternity occurs at rates of 14–18% in wild populations^59,60^. We assigned dummy parents to nestlings with one or two missing parents (*n* = 64), assuming that nestlings within the same clutch were full siblings and thus had the same (dummy) parents. The dummy parents (*n* = 45) were included in the pedigree as founders. The pedigree was ordered using *MasterBayes*^61^ and pruned to only contain informative individuals from the longitudinal TL data set using functions in *MCMCglmm*^62^. The pruned pedigree included 750 individuals (472 maternities and 484 paternities).

### Changes in telomere length with age

We investigated the relationship between TL and age (in days) using within-subject centering^63,64^. This approach allows us to discriminate between effects of age on TL due to selective disappearance at the population level from those due to within-individual TL shortening. For each individual we calculated ∆age by subtracting the individual’s mean age from each sampled age (in days), either with or without log_10_-transformation of age. First, we investigated relationships between TL and age including all TL measurements (*n* = 2977 measurements of *n* = 2667 individuals, excluding 84 individuals with missing sex information), thus, individuals with only one TL measurement (*n* = 2441) had ∆age = 0. We constructed linear mixed-effect models (LMMs) using the *lme4* package^65^ with log_10_-transformed TL as the response variable. Mean age (among-individual effect) and ∆age (within-individual effect) were included as fixed effects covariates. Similar models were fitted with age log_10_-transformed to linearize models. Models including ∆age squared were fitted to account for effects such as a decelerating rate of TL shortening with age. Sex and population identity were included as fixed factors, with individual identity and year included as random intercepts in all models. The five resulting candidate models were compared using Akaike’s information criterion corrected for small sample sizes (AICc^66^). Furthermore, we compared the within- and between-individual effects by including the effect of age instead of ∆age, in which case the effect of mean age represents the difference between the within- and between-subject effects. If the within-individual slope is e.g. more negative than the between-individual slope, this suggests that individuals with short telomeres are more likely to disappear from the population. Models were validated visually using diagnostic plots and all model parameters are reported from models refitted with restricted maximum likelihood (REML). All analyses were performed in R v. 4.2.0^67^.

We have previously found no associations between nestling TL and survival in these sparrow populations, which showed high (presumably extrinsic) juvenile mortality^50^. Effects of selective disappearance of individuals with short TL and/or higher telomere shortening rates could therefore be masked by the majority of individuals having only one early-life TL measurement. We therefore compared relationships between TL and age within and among individuals with multiple TL measurements (*n* = 536 measurements of 226 individuals) using the same procedure described above. The within-individual age effect will be the same in the two approaches, but excluding individuals with only one (early-life) TL measurement (all of which were only sampled as nestlings) allows us to investigate whether selective disappearance may act on TL later in life, as expected if age-dependent TL predicts remaining lifespan^68^.

### Repeatability of telomere length

We used all longitudinal samples (*n* = 536 from 226 individuals) to estimate adjusted individual repeatability^69^ in TL over the lifespan. We used the *rptR* package^70^ to fit a model of variation in log_10_-transformed TL including sex, population identity and log_10_-transformed age (in days) as fixed effects, and year and individual identity as random intercepts. Uncertainty in the estimate was estimated using parametric bootstrap to simulate new data and refit the model for a total of 1000 bootstrap replicates.

### Correlation between early- and later-life telomere length

We tested if the first TL measurement predicted the value of the next subsequently sampled (second) TL measurement (response variable, *n* = 226) using a LMM (*lme4*) with first TL, the elapsed time in days between the two measurements (∆time), sex and population identity as fixed effects, and year as a random intercept. We tested whether including the first TL measurement improved the model using AICc.

In the Supporting Information, we also test for consistent and constant lifelong telomere elongation within individuals with at least three TL measurements (*n* = 61), using the variance-comparison method by Simons et al.^71^ to identify individuals that show increases in TL above what may be expected from measurement error.

### Correlation between the change in telomere length and early-life telomere length

The first TL measurement (‘baseline’ TL) is not statistically independent from the difference between the first and last TL measurement (total ΔTL, *n* = 226) within an individual. Therefore, instead of using ΔTL, we followed Verhulst et al.^33^ in calculating the statistic D by subtracting from ΔTL the change that is expected as a result of this regression-to-the-mean effect, which is estimated from the correlation between the first and last TL measurements. D was multiplied by −1 so that a negative value indicates telomere shortening. We then tested if baseline TL was associated with D using a LMM with D as the response variable and the first TL, the time in days between the first and last TL measurements (∆time), population identity, and sex as fixed effects. Year was included as random intercept. We then tested whether including the first TL measurement improved the model by using AICc.

### Heritabilities of telomere length and the change in telomere length

We constructed univariate Bayesian “animal models”^72^ with either the first early-life TL (*n* = 223) or the last (later-life) TL measurements of an individual as response variables (*n* = 226). Sex, population identity and age of TL measurement (continuous number of days) were included as fixed effects. TL was log_10_-transformed and fitted with a Gaussian error distribution using *MCMCglmm*^62,73^.

We then calculated the difference between the first and last TL measurements (total ΔTL, where negative ΔTL values indicate telomere shortening and positive values indicate lengthening) A third univariate animal model was thus constructed with ΔTL as the response variable (*n* = 226) and ∆time (number of days between the two TL measurements, to account for differences in sampling time intervals), sex and population identity as fixed effects.

For all three models, we estimated variance components for additive genetic effects (‘animal’, *V*_*A*_), brood identity (*V*_*B*_), and hatch year (*V*_*Y*_). Heritabilities were calculated as the proportion of phenotypic variance (*V*_*P*_) explained by additive genetic variance: ${h}^{2}=\frac{{V}_{A}}{{V}_{A}+{{V}_{B}+V}_{Y}+{V}_{R}}$, where *V*_*R*_ is the residual variance. We used inverse-Wishart priors for the random effects and residual variance^73^. The MCMC chain was run for 1,000,000 iterations, sampling every 300 iterations after a burn-in of 10% (100,000 iterations). All autocorrelation values were < 0.05 and effective sample sizes were > 2500. Mixing and stationarity was checked visually and using Heidelberger and Welch's convergence test^74^. Estimates are reported as posterior modes with lower and upper 95% highest posterior density intervals (HPD).

### Factors affecting the change in telomere length

We examined factors affecting the difference between all consecutive pairs of TL measurements (i.e. ΔTL from first to second TL measurement, ΔTL from second to third TL measurement etc., *n* = 301 ΔTL estimates in total) within individuals with multiple TL measurements (*n* = 220 individuals, excluding 6 individuals with missing tarsus length measurements) using LMMs (*lme4*). We fitted sets of candidate models with ΔTL as response variable. As fixed effects, we included combinations of ∆time, age-standardized nestling tarsus length, population identity and sex. As random effects, we included individual identity (to account for multiple ΔTL measurements for individuals with > 2 TL samples, *n* = 61) and hatch year in all models. Brood identity (*n* = 174 broods) explained a negligible amount of variance and was excluded to reduce model complexity and facilitate model convergence. Candidate models were then compared using AICc.

## Results

### Changes in telomere length with age

**Figure 1:**
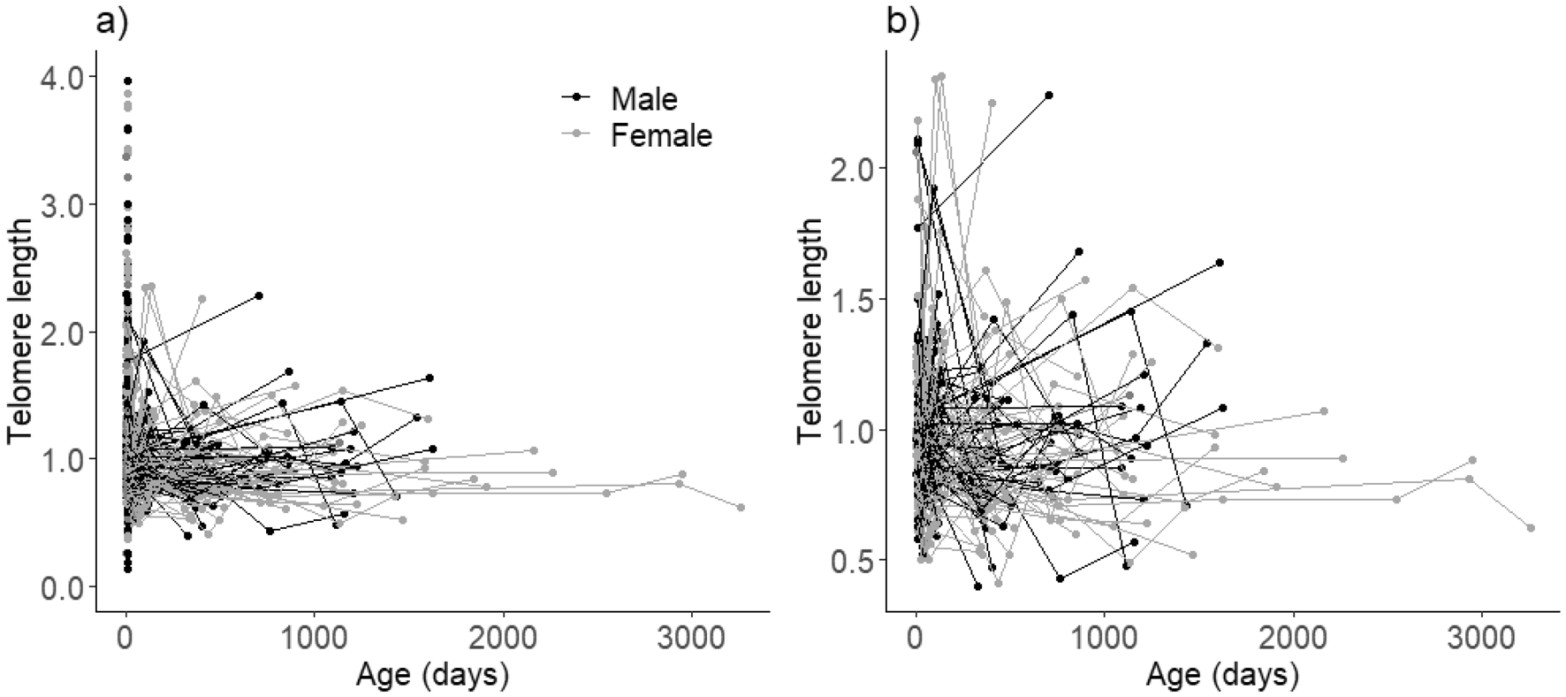
**(a)** Changes in individual telomere length with age in days since hatching in 2667 house sparrows (2977 measurements) sampled across 24 years. Males are shown in black and females in grey. 226 birds were measured at least twice during their lifetime and measurements on the same individual are connected by lines. The oldest sampled individual was 9 years. (**b)** Only individuals with multiple telomere length measurements are shown here for clarity (226 individuals with 536 measurements).

We observed both decreases, stasis, and increases in measured TL with age (Fig. 1 and Fig. S2). After within-subject centering of age, the best model describing the relationship between TL and age including all individuals showed a negative effect of ∆age_log_ (*β*_*∆age_log*_ = −0.019 ± 0.007, CI = [−0.032, −0.006], *n* = 2977, ∆AICc = 1.8 compared to the second-best model, Table 1 and Table S2) and a negative effect of mean age (*β*_*mean_age_log*_ = −0.030 ± 0.006, CI = [−0.043, −0.017]). There was no evidence that these two slopes were different (*β* = −0.011 ± 0.009, CI = [−0.028, 0.007]), suggesting that the decrease in TL with age could be attributed to within-individual telomere shortening.

**Table 1:** Estimates, standard errors (SE), lower and upper 95% confidence intervals (CI) from linear mixed-effects models of variation in telomere length (TL) after within-subject centering of age including all individuals (top) or only individuals with at least two TL measurements (bottom).

	Estimate	SE	Lower CI	Upper CI
All individuals with TL measurements (*n* = 2977)				
Intercept	0.0054	0.0140	*−*0.0219	0.0337
** ∆age**_**log**_	**−0.0194**	**0.0066**	**−0.0324**	**−0.0063**
** Mean age**_**log**_	**−0.0302**	**0.0064**	**−0.0428**	**−0.0174**
Population (Hestmannøy)	0.0096	0.0054	−0.0011	0.0202
Sex (female)	*−*0.0041	0.0041	−0.0123	0.0040
σ^2^_year_ (*n* = 24)	0.0026		0.0013	0.0051
σ^2^_ID_ (*n* = 2667)	0.0014		0.0003	0.0026
σ^2^_residual_	0.0109		0.0097	0.0122
Marginal R^2^/conditional R^2^: 0.011/0.279				
Only individuals with multiple TL measurements (*n* = 536)				
** Intercept**	**−0.0702**	**0.0291**	**−0.1275**	**−0.0135**
** ∆age**_**log**_	**−0.0158**	**0.0070**	**−0.0295**	**−0.0019**
Mean age_log_	0.0092	0.0155	−0.0209	0.0398
Population (Hestmannøy)	0.0277	0.0183	−0.0079	0.0635
Sex (female)	−0.0103	0.0109	−0.0316	0.0112
σ^2^_year_ (*n* = 22)	0.0010		0.0000	0.0027
σ^2^_ID_ (*n* = 226)	0.0013		0.0003	0.0028
σ^2^_residual_	0.0121		0.0104	0.0141
Marginal R^2^/conditional R^2^: 0.018/0.175				

The models included individual identity and year as random intercepts. Bold indicates parameters with CIs not overlapping zero.

When only including individuals with longitudinal (multiple) TL measurements (Fig. 1b) the composition of the best model was identical to the above, but the among individual effect of mean age was now uncertain and close to zero (*β*_*mean_age_log*_ = 0.009 ± 0.016, CI = [−0.021, 0.040]), ∆AICc = 1.3, Table 1 and Table S3). We thus found some evidence for a difference between the within- and among-individual effects (*β* = 0.025 ± 0.017, CI = [−0.008, 0.058], with a CI overlapping zero), which indicates selective disappearance of birds with short TL and/or faster telomere attrition rates through life.

### Repeatability of telomere length

The adjusted repeatability of log_10_-transformed TL was found to be 0.092 ± 0.049 (CI = [0.000, 0.194]), which means that 9.2% of the variation in longitudinal TL measurements was explained by within-individual consistency.

### Correlation between early- and later-life telomere length

**Figure 2:**
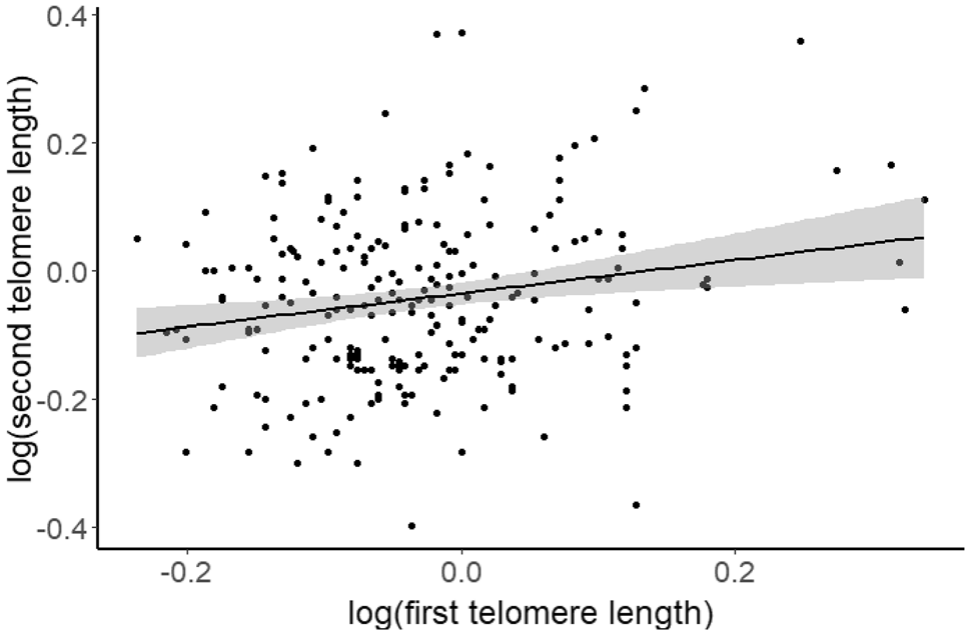
First telomere length mainly measured in nestlings plotted against the second telomere length measured in later-life (juvenile or adult) in house sparrows (*n* = 226). The regression line (black) reflects the estimate from Table 2 and 95% confidence intervals are shown in grey.

The average follow-up time (∆time) from the first to the second sampled TL measurement was 269 ± 370 SD days. Including the first TL measurement improved the model explaining variation in the second (subsequent) TL measurement (∆AICc = 4.9). There was a positive association between the first and second TL measurements (*β*_*log10(firstTL)*_ = 0.234 ± 0.089, CI = [0.062, 0.408], Table 2 and Fig. 2). This means that individuals with long early-life TL also had long later-life TL, but with considerable individual variation (Fig. 2).

**Table 2:** Estimates, standard errors (SE), lower and upper 95% confidence intervals (CI) from a linear mixed-effects model of variation in the second telomere length (TL) measurement (*n* = 226) in two populations of house sparrows.

Response variable: log_10_(second TL)	Estimate	SE	Lower CI	Upper CI
**Intercept**	**−0.0974**	**0.0272**	**−0.1501**	**−0.0448**
**log**_**10**_**(first TL)**	**0.2342**	**0.0888**	**0.0625**	**0.4077**
∆time	**−**0.18E**−**5	2.4E**−**5	**−**4.9E**−**5	4.4E**−**5
**Population (Hestmannøy)**	**0.0633**	**0.0254**	**0.0142**	**0.1130**
Sex (female)	0.0048	0.0167	**−**0.0278	0.0374
σ^2^_year_ (*n* = 18)	0.0012		8.2E**−**5	0.0036
σ^2^_residual_	0.0155		0.0124	0.0180
Marginal R^2^/conditional R^2^: 0.062/0.129				

The model included year as random intercept. Bold indicates parameters with CIs not overlapping zero.

### Correlation between the change in telomere length and early-life telomere length

The average follow-up time (∆time) from the first to the last TL measurement was 345 ± 406 SD days. After correcting for regression-to-the-mean effects, including the first TL measurement in the model describing variation in D (i.e. corrected ∆TL) did not improve the model (∆AICc = −1.7) and therefore there was no evidence for a dependency of telomere attrition on early-life TL (*β*_*firstTL*_ = 0.052 ± 0.077, CI = [−0.099, 0.201]).

### Heritabilities of telomere length and the change in telomere length

The three univariate animal models (Table 3) revealed additive genetic variances (*V*_*A*_) for early-life TL (*V*_*A*_ = 0.0182, HPD = [0.0131, 0.0235]), later-life TL (*V*_*A*_ = 0.0205, HPD = [0.0150, 0.0272]), and ∆TL (*V*_*A*_ = 0.0531, HPD = [0.0343, 0.0838]). The heritabilities were similar for early-life TL (*h*^*2*^ = 0.1358, HPD = [0.0804, 0.2090]) and later-life TL (*h*^2^ = 0.1542, HPD = [0.0917, 0.2268]), but somewhat higher for ∆TL (*h*^2^ = 0.2121, HPD = [0.1194, 0.3153]). The main source of variation in all three TL traits was hatch year, explaining 60% of the variation in early-life TL, 53% in later-life TL, and 37% in ∆TL. Brood effects explained 14–16% of the variation in each TL trait.

**Table 3:** Posterior modes and lower and upper 95% highest posterior density intervals (HPD) for fixed effects, variance components, and heritability estimates from three univariate animal models of the variation in early-life telomere length (TL, first measurement, log_10_-transformed, *n* = 223), later-life TL (last measurement, log_10_-transformed, *n* = 226), and the change between first and last TL measurements (∆TL, *n* = 226), respectively, in two populations of house sparrows.

Variable	log_10_(first TL)			log_10_(last TL)			∆TL		
Fixed effects	Estimate	HPD		Estimate	HPD			HPD	
		Lower	Upper		Lower	Upper	Estimate	Lower	Upper
Intercept	−0.0008	−0.2518	0.2618	−0.1145	−0.3072	0.0581	−0.1258	−0.3882	0.1261
Sex (female)	−0.0202	−0.0787	0.0370	0.0202	−0.0357	0.0873	0.0938	0.0003	0.2013
Island identity (Hestmannøy)	0.0032	−0.1108	0.1255	0.0373	−0.0836	0.1580	0.0674	−0.1586	0.2359
Age (days)	0.0037	−0.0197	0.0141	1.7E−5	−4.3E−5	0.0001	–	–	–
∆time (days)	–	–	–	–	–	–	8.5E−6	−0.0001	0.0002
**Variance components**									
*h*^2^	0.1358	0.0804	0.2090	0.1542	0.0917	0.2268	0.2121	0.1194	0.3153
*V*_*A*_	0.0182	0.0131	0.0235	0.0205	0.0150	0.0272	0.0531	0.0343	0.0838
*V*_*B*_	0.0189	0.0135	0.0245	0.0195	0.0140	0.0268	0.0427	0.0303	0.0666
*V*_*Y*_	0.0788	0.0332	0.1398	0.0658	0.0358	0.1418	0.0926	0.0437	0.1997
*V*_*R*_	0.0165	0.0120	0.0210	0.0180	0.0137	0.0242	0.0596	0.0379	0.0824

*h*^*2*^ heritability, *V*_*A*_ additive genetic variance, *V*_*B*_ brood variance, *V*_*Y*_ year variance, *V*_*R*_ residual variance.

### Factors affecting the change in telomere length

**Figure 3:**
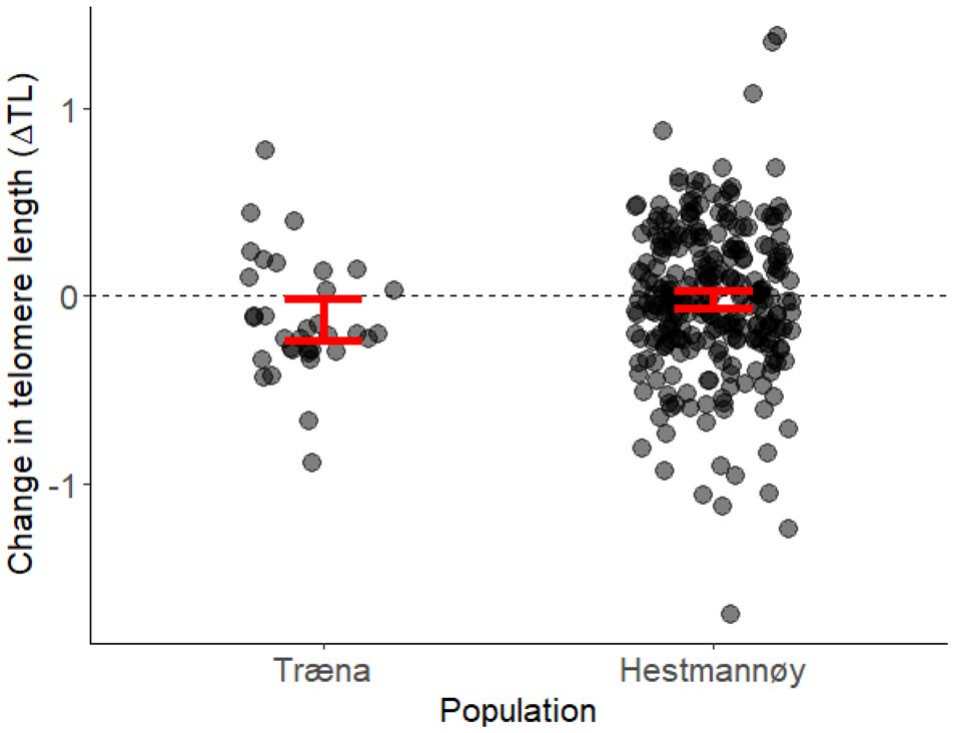
The change in telomere length (∆TL) across all measures of TL changes (*n* = 310 in total, *n* = 276 from Hestmannøy and *n* = 34 from Træna) within 226 individuals in two house sparrow populations. Negative ∆TL values indicate telomere shortening and positive ∆TL values indicate telomere lengthening. Red bars show 95% confidence intervals based on the t-distribution around the sample means.

The model describing variation in all ΔTL measurements that only included the intercept was almost indistinguishable (∆AICc = 0.2) from the highest ranked model (Table S4). The highest ranked model included population identity, which indicated a tendency towards higher ΔTL in the Hestmannøy population (*β*_*population[Hestmannøy]*_ = 0.105 ± 0.072, CI = [−0.034, 0.248]), but with a CI overlapping zero. That is, individuals in the Hestmannøy population may tend to experience less telomere shortening over time than individuals from the Træna population (Fig. 3).

## Discussion

In accordance with many, but not all, studies on animals^22,75,76^ we found evidence for age-related telomere shortening within house sparrows (Fig. 1 and Table 1), as expected given the somatic costs associated with biological ageing and cumulative stress experiences (e.g.^15,17^). In common with other non-mammalian vertebrates, birds have nucleated erythrocytes. Therefore, TLs derived from whole blood samples are mainly measured in erythrocytes, which are normally produced in the bone marrow^77^. Compared to other tetrapods, avian erythrocytes have a relatively short lifespan of 1 month in vivo (28 days in house sparrows^78^) with ~ 3% being replaced each day^79^. Thus, we may expect to observe changes in blood TL in house sparrows over periods within days or weeks, while other less proliferative tissues may experience substantially less TL attrition^80^.

The low within-individual repeatability in TL measurements observed in this study (9.2%) was similar to other qPCR studies with large sample sizes^26,81–83^ and reflects the consistency in TL within individuals over the lifespan. Some studies using the TRF method have reported higher repeatabilities than qPCR studies (in different species, e.g.^47,68,84^), which may in part be attributed to shorter follow-up times and higher measurement error of qPCR, which will decrease repeatability^85^. We may expect low TL repeatability when including early-life stages when telomere shortening rates are expected to be most variable (^85^, but see^22^). Nevertheless, the first TL measurement predicted subsequent TL measurements within individuals, with individuals with a short early-life TL having a short TL later in life (Fig. 2). This suggests that the negative effects of growth^48^, environmental stressors^50^ and inbreeding^86^ on early-life TL previously described in these populations may have lasting effects on TL later in life^19,84^. Recent studies have found a positive genetic correlation close to 1 between TL measurements within individuals, suggesting that the same genes are involved in controlling TL at different ages^32,42,47^. However, our sample size was smaller than previous studies and we lack the sufficient power to estimate such genetic correlations with high precision and accuracy (e.g.^87^).

The heritability estimate for ∆TL (*h*^2^ = 0.21) was higher than that reported for ∆TL in western jackdaws (*h*^2^ = 0.09^47^), but in our study the follow-up times across all TL measurements (∆time) were much longer and more variable (25 days in Bauch et al.^47^ vs. 343 ± 410 SD days in this study). Correspondingly, heritability of TL shortening in humans was found to be even higher (*h*^2^ = 0.28^45^) in a study that had even longer follow-up times (on average 12 years). We found a considerable effect of hatch year, which explained 37% of the variance in ∆TL (vs. 4% in^47^). This may reflect annual environmental variation experienced by different cohorts in early-life such as weather conditions and competition^43,50^ and suggests that there are persistent impacts of the early-life environment on TL shortening later in life^19^.

The heritability estimates for early-life TL and later-life TL were of similar magnitude (*h*^2^ ~ 0.15, Table 3), but much smaller than in the jackdaw and human studies^45,47^. We previously estimated the early-life TL heritability for a much larger sample of nestlings from the same populations to be smaller (*h*^2^ = 0.04, *n* = 2662^43^). However, the sample in the present study only included individuals surviving until the time of the second TL measurement (as juveniles or adults). This may bias the TL heritability estimates if individuals are not missing at random with respect to the trait of interest^88^; e.g. if mortality (and hence missingness) depends on TL^21^, the distributional properties of the sampled individuals may differ from the whole population and lead to biased inferences. It is tempting to suggest that the lower heritability estimates of TL compared to ∆TL reflects a closer association between fitness and TL or the environmental conditions that TL reflects (e.g.^89^). Indeed, TL may be unlikely to become critically short in house sparrows^48^, and the early-life environment has strong influences on both TL and ∆TL, as shown in this study.

We have previously found some evidence for a negative association between early-life TL and annual reproductive success in house sparrows^50^. We speculated that telomere shortening later in life depended upon early-life TL to explain this pattern. However, in this study we found no evidence that early-life TL was associated with telomere shortening rates (when correcting for regression-to-the-mean—see “Methods”). Thus, individuals with short early-life TL may indeed exhibit a faster life-history involving a higher reproductive output and lower somatic maintenance^50,90,91^. In this study, we found weak evidence for selective disappearance of individuals (that survived fledgling and/or juvenile stages) with short telomeres (or faster telomere attrition rates), which has been observed in longitudinal studies in several species of wild birds and mammals^27,32,68,82,83,92^. Thus, TL measured in adulthood, or telomere attrition rate^40^, but not early-life TL^50^, may reveal the expected relationships between telomere dynamics and mortality^21^, but future studies are needed that more comprehensively investigate the associations between TL, ∆TL and fitness components.

House sparrows on the island of Træna tended to experience greater telomere shortening (i.e. more negative values of ∆TL) than those on Hestmannøy (Fig. 3), but the evidence for this effect was weak as the more parsimonious intercept model was almost indistinguishable from the highest ranked model (Table S4). Individuals experiencing more stressful conditions, such as harsh abiotic conditions, competition, parasite infection, anthropogenic effects and/or poor diet, have been shown to exhibit increased rates of telomere shortening in several species^17^. We have previously shown that early-life TL in nestling sparrows on Træna was more negatively affected by higher conspecific population densities than in the Hestmannøy population^50^. In line with this, we now find that the Træna population overall tends to exhibit higher rates of telomere shortening. However, further studies on multiple populations are required to disentangle the specific (environmental) effects shaping such population differences^93^. Contrasting intraspecific TL dynamics have also been found in different populations of European roe deer (*Capreolus* *capreolus*) whose habitats differ in food availability^94^, in great tits (*Parus* *major*) living in urban or rural environments where diet composition differs^95^, in American redstarts (*Setophaga* *ruticilla*) overwintering in different non-breeding habitat types that also vary in food availability^96^, in pied flycatchers (*Ficedula* *hypoleuca*) breeding in different habitats across Europe^97^, and in populations of spotted snow skinks (*Niveoscincus* *ocellatus*^98^), common lizards (*Zootoca* *vivipara*^99^) and moose (*Alces* *alces*^100^) experiencing different thermal environments.

Our study highlights the plastic nature of telomere length, which may both shorten and lengthen with time within individuals (e.g.^101–103^). Telomere lengthening has been thought to represent measurement error^104^, but recent studies have produced evidence that telomere lengthening occurs in several species^25,26,36,71,83,105–108^. Telomeres may lengthen due to the activity of the enzyme telomerase^109^ and other mechanisms (e.g.^110,111^). We identified one individual (a female from the Hestmannøy population) that showed significant consistent telomere elongation throughout life at a greater rate than might have been expected by measurement error (see Supporting Information). Recent studies on house sparrows in other populations have also showed instances of telomere lengthening within some individuals^90,112^ and that house sparrows may experience rather transient TL shortening in response to stressors^113^. However, TL also generally declined with age in another long-term study of house sparrows^22,90^.

Somatic telomerase activity has been detected in tissues of some species, including birds^114^, but is generally thought to be repressed in large bodied and long-lived species as a mechanism of tumor suppression^115,116,117^. However, little is known about the energetic costs of TL maintenance^16,118,119^, and telomerase activity and telomere maintenance are not well-known within house sparrows. For instance, cycloastrogenol (TA‐65^120^), which activates telomerase and lengthens telomeres in blood in mice (*Mus* *musculus*^121^), humans (*Homo* *sapiens*^122^), zebra finches (*Taenopygia* *guttata*^123^), and tree swallows (*Tachycineta* *bicolor*^124^) was found to shorten telomeres in blood in house sparrow fledglings^125^. Experimental manipulations of TL or telomerase activity^123,126,127^ may be necessary to further our understanding of the causal role of telomere dynamics in shaping organismal life-histories^119^.

In conclusion, we found evidence of general telomere shortening with age within individuals, but also several instances of apparent telomere lengthening and at least one case of consistent lengthening through life in wild house sparrows. Early-life TL predicted later-life TL, but the change in TL was independent of early-life TL. There was a moderate heritability of ∆TL, which was higher than the heritability of TL, but most of the variation in both ∆TL and TL was explained by cohort effects. Furthermore, we found indications of population differences in ∆TL that may be linked to habitat differences. Combined, our study indicates that telomere dynamics are influenced by both genetic and environmental variation, and that TL may be more phenotypically flexible within individuals than previously anticipated.

## Supplementary Information

Supplementary Information.

## Data Availability

Data is available on the Open Science Framework (OSF) https://doi.org/10.17605/OSF.IO/4CJ3S.
