# Supplementary material for: Longitudinal telomere dynamics within natural lifespans of a wild bird
Source: Sci Rep. 2023 Mar 15;13:4272. doi: 10.1038/s41598-023-31435-9 (PMC10017829; doi:10.1038/s41598-023-31435-9)
Supplement: Supplementary file 1 — Supplementary Information. [file 41598_2023_31435_MOESM1_ESM.docx]

*Supporting information*

**Longitudinal telomere dynamics within natural lifespans of a wild bird**

Michael Le Pepke^*1^, Thomas Kvalnes^1^, Jonathan Wright^1^, Yimen G. Araya-Ajoy^1^, Peter Sjolte Ranke^1^, Winnie Boner^2^, Pat Monaghan^2^, Bernt-Erik Sæther^1^, Henrik Jensen^1^ & Thor Harald Ringsby^1^

^1^Centre for Biodiversity Dynamics (CBD), Department of Biology, Norwegian University of Science and Technology (NTNU), Trondheim, Norway

^2^School of Biodiversity, One Health and Veterinary Medicine, University of Glasgow, Glasgow, UK

^*^Correspondence: Michael Le Pepke, email: michael@pepke.dk

**Contents**

[Sample sizes 2](#_Toc113794179)

[Distribution of the age of individuals when telomere length is measured 3](#_Toc113794180)

[Distribution of changes in telomere length 3](#_Toc113794181)

[Testing for consistent telomere elongation within individuals 4](#_Toc113794182)

[AICc table of models describing variation in TL as a function of age 4](#_Toc113794183)

[AICc table of models describing variation in ∆TL 5](#_Toc113794184)

[References 6](#_Toc113794185)

## **Sample sizes**

**Table S1:** Numbers (sample sizes) of telomere length sampled fledglings and (re-)captured juveniles or adults for each cohort (year) and island (Hestmannøy and Træna) used in this study. The same individual may have been recaptured more than once, but in different years.

| **Cohort /year** | **Hestmannøy**  **number of fledglings** | **Hestmannøy**  **number of juveniles/ adults** | **Træna number of fledglings** | **Træna number of juveniles/ adults** | **Total number of fledglings** | **Total number of samples** |
| --- | --- | --- | --- | --- | --- | --- |
| **1994** | 103 | 2 | - | - | 103 | 105 |
| **1995** | 90 | 2 | - | - | 90 | 92 |
| **1996** | 48 | 0 | - | - | 48 | 48 |
| **1997** | 81 | 2 | - | - | 81 | 83 |
| **1998** | 100 | 1 | - | - | 100 | 101 |
| **1999** | 90 | 0 | - | - | 90 | 90 |
| **2000** | 73 | 0 | - | - | 73 | 73 |
| **2001** | 41 | 0 | - | - | 41 | 41 |
| **2002** | 97 | 2 | - | - | 97 | 99 |
| **2003** | 106 | 0 | - | - | 106 | 106 |
| **2004** | 106 | 0 | 67 | 0 | 173 | 173 |
| **2005** | 95 | 0 | 90 | 0 | 185 | 185 |
| **2006** | 123 | 24 | 59 | 7 | 182 | 213 |
| **2007** | 152 | 29 | 127 | 13 | 279 | 321 |
| **2008** | 83 | 19 | 38 | 7 | 121 | 147 |
| **2009** | 182 | 31 | 35 | 6 | 217 | 254 |
| **2010** | 129 | 39 | 44 | 1 | 173 | 213 |
| **2011** | 248 | 37 | 65 | 0 | 313 | 350 |
| **2012** | 70 | 23 | 24 | 0 | 94 | 117 |
| **2013** | 93 | 44 | 87 | 0 | 180 | 224 |
| **2014** | - | 14 | - | 0 | - | 14 |
| **2015** | - | 7 | - | 0 | - | 7 |
| **2016** | - | 2 | - | 0 | - | 2 |
| **2017** | - | 3 | - | 0 | - | 3 |
| **Sum:** | 2110 | 281 | 636 | 34 | 2746 | 3061 |

##
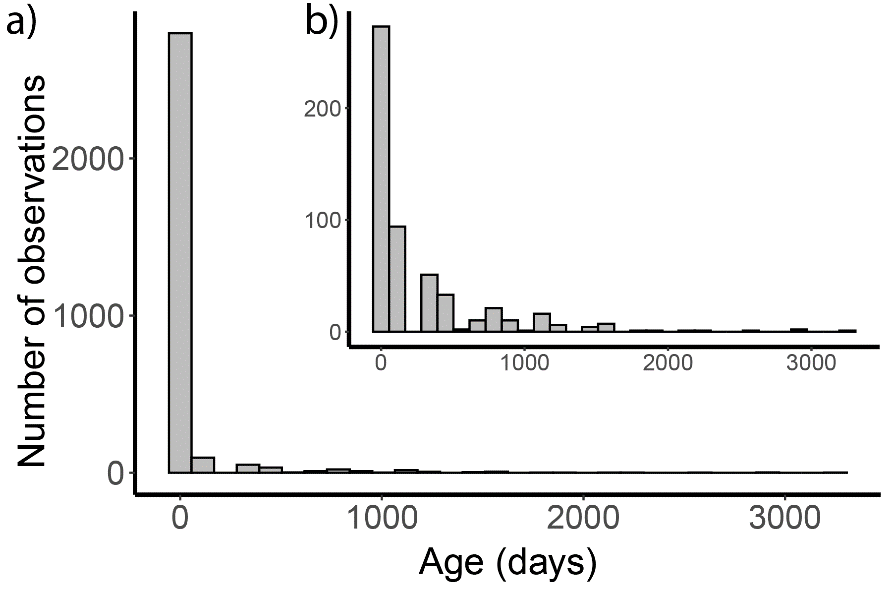
**Distribution of the age of individuals when telomere length is measured**

**Fig. S1:** Histogram of the age of individuals when telomere length is measured including a) all individuals (*n*=2977 measurements) or b) only individuals with multiple telomere length measurements (*n*=536 measurements).

## **Distribution of changes in telomere length**


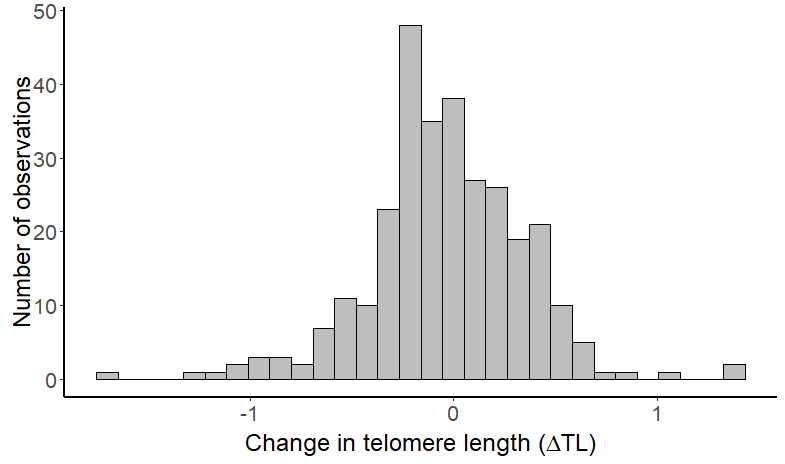
 **Fig. S2:** Histogram of all observed changes in telomere length (∆TL, *n*=310) including multiple ∆TL per individual (*n*=226). If we define a change in |∆TL|<0.1 as stasis, then 41% of the observations were decreases in TL, 35% were increases in TL, and 24% were stasis.

## **Testing for consistent telomere elongation within individuals**

We observed both increases and decreases in TL with age within individuals (Fig. 1). Telomere elongation has been thought to represent measurement error, but it may also be a true change in TL (e.g. Bateson & Nettle, 2017). For individuals with at least 3 TL measurements we can test for consistent telomere elongation through individual lifetimes following the approach by Simons et al. (2014). They assumed that increases in TL that are significantly greater than the within-individual variance in TL may represent (true) consistent TL elongation. We used the first, second and last TL measurements of all individuals with at least 3 TL measurements (*n*=61). We applied equation 7 in Simons et al. (2014) to identify individuals that showed TL increases greater than that expected by measurement error (with 95% confidence). This approach identified one female (ID: 8L89211) from the Hestmannøy population that experienced a TL increase from first (10 days old in year 2008) to second (119 days old) measurements of 23% and from second to last (390 days old) measurements of 91%. This individual was not observed after 2009 and is thus likely to have died in its second winter. Thus, while TL may both increase and decrease within the same individuals, at least some individuals may show consistent TL lengthening through life. We note that the test by Simons et al. (2014) assumes that the rate of TL change is constant over time (Nettle & Bateson, 2017). Thus, individuals with telomere lengthening rates that vary over time (e.g. from year to year) will not be detected as true lifetime telomere elongation using this test.

## **AICc table of models describing variation in TL as a function of age**

**Table S2:** AICc table of candidate models describing variation in TL as a function of age including all TL measurements (*n*=2977) after within-subject centering. ∆age and mean age were calculated either before (∆age_lin_ and mean age_lin_) or after log_10_-transformation of age (∆age_log_ and mean age_log_). All models included individual identity and year as random intercepts. Models are ranked by AICc and number of degrees of freedom (df) and model weights (w) are reported.

|  | Model (*n*=2977) | ∆AICc | df | w |
| --- | --- | --- | --- | --- |
| 1 | log(TL) = sex + population + ∆age_log_ + mean age_log_ | 0.0 | 8 | 0.710 |
| 2 | log(TL) = sex + population + ∆age_log_ + ∆age_log_^2^ + mean age_log_ | 1.8 | 9 | 0.290 |
| 3 | log(TL) = sex + population + ∆age_lin_ + mean age_lin_ | 17.7 | 8 | <0.01 |
| 4 | log(TL) = sex + population + ∆age_lin_ + ∆age_lin_^2^ + mean age_lin_ | 19.3 | 9 | <0.01 |
| 5 | log(TL) = sex + population | 24.5 | 6 | <0.01 |

**Table S3:** AICc table of candidate models describing variation in TL as a function of age including only individuals with at least two TL measurements (*n*=536) after within-subject centering. ∆age and mean age were calculated either before (∆age_lin_ and mean age_lin_) or after log_10_-transformation of age (∆age_log_ and mean age_log_). All models included individual identity and year as random intercepts. Models are ranked by AICc and number of degrees of freedom (df) and model weights (w) are reported.

|  | Model (*n*=536) | ∆AICc | df | w |
| --- | --- | --- | --- | --- |
| 1 | log(TL) = sex + population + ∆age_log_ + mean age_log_ | 0.0 | 8 | 0.440 |
| 2 | log(TL) = sex + population | 1.3 | 6 | 0.234 |
| 3 | log(TL) = sex + population + ∆age_log_ + ∆age_log_^2^ + mean age_log_ | 1.8 | 9 | 0.178 |
| 4 | log(TL) = sex + population + ∆age_lin_ + mean age_lin_ | 2.9 | 8 | 0.105 |
| 5 | log(TL) = sex + population + ∆age_lin_ + ∆age_lin_^2^ + mean age_lin_ | 4.6 | 9 | 0.043 |

## **AICc table of models describing variation in ∆TL**

**Table S4:** AICc table of candidate models describing variation in ∆TL (*n*=301). All models included individual identity and year as random intercepts. Models are ranked by AICc and number of degrees of freedom (df) and model weights (w) are reported.

|  | Model (*n*=296) | ∆AICc | df | w |
| --- | --- | --- | --- | --- |
| 1 | ∆TL = population | 0.0 | 5 | 0.199 |
| 2 | ∆TL = (intercept) | 0.2 | 4 | 0.184 |
| 3 | ∆TL = ∆time | 1.7 | 5 | 0.085 |
| 4 | ∆TL = population + sex | 1.9 | 6 | 0.078 |
| 5 | ∆TL = population + ∆time | 1.9 | 6 | 0.077 |
| 6 | ∆TL = population + tarsus | 2.0 | 6 | 0.074 |
| 7 | ∆TL = sex | 2.0 | 5 | 0.072 |
| 8 | ∆TL = tarsus | 2.1 | 5 | 0.071 |
| 9 | ∆TL = sex + ∆time | 3.6 | 6 | 0.033 |
| 10 | ∆TL = tarsus + ∆time | 3.7 | 6 | 0.031 |
| 11 | ∆TL = population + sex + ∆time | 3.8 | 7 | 0.030 |
| 12 | ∆TL = population + sex + tarsus | 3.9 | 7 | 0.029 |
| 13 | ∆TL = sex + tarsus | 3.9 | 6 | 0.028 |
| 14 | ∆TL = population + sex + tarsus + ∆time | 5.9 | 8 | 0.011 |

## **References**

Bateson, M., & Nettle, D. (2017). The telomere lengthening conundrum – it could be biology. *Aging Cell, 16*(2), 312-319. doi:10.1111/acel.12555

Nettle, D., & Bateson, M. (2017). Detecting telomere elongation in longitudinal datasets: analysis of a proposal by Simons, Stulp and Nakagawa. *PeerJ, 5*, e3265. doi:10.7717/peerj.3265

Simons, M. J. P., Stulp, G., & Nakagawa, S. (2014). A statistical approach to distinguish telomere elongation from error in longitudinal datasets. *Biogerontology, 15*(1), 99-103. doi:10.1007/s10522-013-9471-2
